# Supplementary material for: Telemedicine adoption in cardiology: Determinants and predictors identified using Bayesian Model Averaging and Machine Learning
Source: PLOS Digit Health. 2026 Apr 20;5(4):e0001359. doi: 10.1371/journal.pdig.0001359 (PMC13095100; doi:10.1371/journal.pdig.0001359)
Supplement: S1 Table — Complete list of all 34 items included in the pre-validated web-based survey assessing knowledge, acceptance, and utilization of telemedicine among healthcare professionals in cardiology care. (DOCX) [file pdig.0001359.s001.docx]

**S1 Table:** Full list of questionnaire items

| **No.** | **Item** | **Response options** | **Response format** | **Filter condition** | **Variable name(s)** |
| --- | --- | --- | --- | --- | --- |
| **Section 1: Telemedicine – Use & Experiences** | | | | | |
| Q1 | How would you rate your knowledge of telemedicine? | Very poor; Poor; Partly good/partly poor; Good; Very good | 5-point Likert (single choice) |  | TM_know |
| Q2 | Do you already use telemedicine? | Daily; Approx. 3–4×/week; Approx. 3–4×/month; Approx. 3–4×/quarter; Not at all | Ordinal (single choice) |  | TM_use |
| Q3 | In what form do you use telemedicine? | Video consultation; Telephone consultation; Telemonitoring; Apps (e.g. DiGAs); Home monitoring (implants); Wearables; Other | Multiple choice | Q2 ≠ 'Not at all' | — |
| Q4 | For what reasons do you use telemedicine? (Please prioritise: 1, 2, 3…) | Infection control; Modern/future-oriented practice; Time savings; Overcoming distances; Patient request; Positive evidence base; Economic benefit; Competitive advantage; Other | Multiple choice + prioritisation comment | Q2 ≠ 'Not at all' | — |
| Q5 | When do you predominantly use telemedicine? (percentages must sum to 100%) | During working hours (%); Outside working hours (%) | Open numeric | Q2 ≠ 'Not at all' | — |
| Q6 | From where do you mostly use telemedicine? | From home; From practice/hospital; Other | Single choice | Q2 ≠ 'Not at all' | — |
| Q7 | From which device do you predominantly use telemedicine? | Professional device; Personal device | Single choice | Q2 ≠ 'Not at all' | — |
| Q7a | Which personal device? | Smartphone; Tablet; PC; Other | Single choice | Q7 = 'Personal device' | — |
| Q7b | Which professional device? | Smartphone; Tablet; Clinic/practice PC; Other | Single choice | Q7 = 'Professional device' | — |
| Q10 | Would you like to expand your use of telemedicine in the future? | Yes, definitely; Yes; Don't know; No; Definitely not | 5-point Likert (single choice) | Q2 ≠ 'Not at all' | — |
| Q11 | Would you like to use telemedicine approaches? | Yes, definitely; Yes; Don't know; No; Definitely not | 5-point Likert (single choice) | Q2 = 'Not at all' | — |
| Q12 | Which telemedicine approaches would you like to use? | Video consultation; Telephone consultation; Telemonitoring; Apps (e.g. DiGAs); Home monitoring (implants); Wearables; Other | Multiple choice | Q11 = 'Yes' or 'Yes, definitely' | — |
| **Section 2: Telemedicine – Use & Experiences II** | | | | | |
| Q13 | Is there anything stopping you from using telemedicine? | Yes; Rather yes; Don't know; Rather no; No | 5-point Likert (single choice) |  | — |
| Q14 | What is stopping you from using telemedicine? | Cost of equipment; Poor internet connection; Administrative burden; No reimbursement possible; Insufficient reimbursement structure; Data security; Insufficient evidence on patient benefit; Other | Multiple choice | Q13 = 'Yes' or 'Rather yes' | — |
| Q15 | Between which actors should telemedicine-based exchange take place? | Physician–Physician; Physician–Medical assistant; Physician–Patient; Other (specify); No exchange desired | Single choice + open comment |  | — |
| Q16 | Which cardiological conditions are particularly suitable for telemedicine? | — | Open text |  | — |
| Q17 | Areas in cardiological care where telemedicine is particularly relevant? | Basic monitoring (weight, BP, ECG); Extension of follow-up intervals; Acute events (e.g. ECG in MI); Other | Multiple choice |  | — |
| Q18 | Areas in cardiological care where telemedicine is less suitable? | Basic monitoring (weight, BP, ECG); Extension of follow-up intervals; Acute events (e.g. ECG in MI); Other | Multiple choice |  | — |
| Q19 | What structural/organisational/institutional conditions should be created for telemedicine in Brandenburg? | — | Open text |  | — |
| Q20 | How do you assess the need for continuing education on telemedicine among professional colleagues? | Very high; High; Neutral; Low; Very low | 5-point Likert (single choice) |  | assess_relev |
| Q21 | How do you assess colleagues' willingness to undertake continuing education on telemedicine? | Very high; High; Neutral; Low; Very low | 5-point Likert (single choice) |  | assess_willing |
| Q22 | How high is your own willingness to participate in continuing education on telemedicine? | Very high; High; Neutral; Low; Very low | 5-point Likert (single choice) |  | TM_course |
| Q23 | Would you be willing to invest (financially) in telemedicine for your everyday care? | High willingness; Moderate willingness; Neutral; Moderate reluctance; Strong reluctance | 5-point Likert (single choice) |  | invest |
| Q24 | Under what conditions would you invest in telemedicine applications? | — | Open text | Q23 = 'Neutral', 'Moderate' or 'High willingness' | — |
| Q25 | Do you use telemedicine applications privately for your own health? | Yes, often; Yes, sometimes; Don't know; Not yet; No, and I don't intend to | Single choice |  | TM_use_perso |
| Q26 | If yes, which telemedicine applications do you use privately? | — | Open text | Q25 = 'Yes, often' or 'Yes, sometimes' | — |
| **Section 3: Sociodemographic Data** | | | | | |
| Q27 | Age | < 20; 20–29; 30–39; 40–49; 50–59; 60–69; 70–79; ≥ 80 | Ordinal (single choice) |  | age |
| Q28 | Gender | Male; Female; Diverse | Single choice |  | — |
| Q29 | Medical specialty | Internal medicine; Internal medicine and cardiology; General medicine; Other specialty; Not yet a specialist | Single choice |  | — |
| Q30 | Primary area of medical care | Outpatient; Inpatient | Single choice |  | prime_care |
| Q31 | Type of organisation | Solo practice; Group practice; Medical care centre (MVZ); Polyclinic; Other | Single choice | Q30 = 'Outpatient' | — |
| Q32 | Number of hospital beds | — | Open numeric | Q30 = 'Inpatient' | — |
| Q33 | Position at hospital | Medical director; Chief physician; Senior physician; Specialist physician; Resident physician | Single choice |  | — |
| Q34 | Location classification | Large city (> 100,000); Medium-sized city (20,000–100,000); Small town (5,000–20,000); Rural community (< 5,000) | Single choice |  | — |
| Q35 | Average number of patients per quarter | — | Open numeric |  | nb_patient |
| **Section 4: Final Comments** | | | | | |
| Q36 | Do you have any further comments on the questionnaire or the topic of telemedicine? | — | Open text |  | — |

BP = blood pressure; DiGA = Digitale Gesundheitsanwendung (digital health application); ECG = electrocardiogram; MI = myocardial infarction; MVZ = Medizinisches Versorgungszentrum (medical care centre). Filter condition: items were only displayed to respondents meeting the specified routing criteria. Response options for open-text, open-numeric items or anonymized items are marked with a dash (—). Variable names refer to dataset column labels. Item numbering follows the original German instrument; some numbers are skipped due to branching logic.
