# Supplementary material for: Telemedicine adoption in cardiology: Determinants and predictors identified using Bayesian Model Averaging and Machine Learning
Source: PLOS Digit Health. 2026 Apr 20;5(4):e0001359. doi: 10.1371/journal.pdig.0001359 (PMC13095100; doi:10.1371/journal.pdig.0001359)
Supplement: S2 Table — Determinants of telemedicine use identified through Bayesian model averaging after exclusion of participants with missing values, including posterior inclusion probabilities and coefficient estimates. (DOCX) [file pdig.0001359.s002.docx]

**S3 Table:** Factors Identified Through BMA and Their Association With TM Use for the sensitivity analysis

| **Determinants** | **Top**  **model** | **Top 5**  **models** | **BMA** | | **Posterior**  **inclusion  probability (%)** | **Direction** |
| --- | --- | --- | --- | --- | --- | --- |
|  | mean | mean | mean | sd |  |  |
| TM knowledge | 1.59 | 1.00 | 1.59 | 0.49 | 100 | Positive association |
| TM barrier: current remuneration structure insufficient | 0 | 0 | 0.07 | 0.31 | 7.70 | Positive association |
| TM barrier: insufficient data for the benefit of patients | 0 | 0.12 | -0.43 | 0.96 | 22.1 | Negative association |
| TM use suitable for heart failure | 1.02 | 1.00 | 1.02 | 1.03 | 58.2 | Positive association |
| TM use suitable for monitoring an event | 1.74 | 1.00 | 1.74 | 1.00 | 86.5 | Positive association |
| TM use less suitable for extension of the aftercare interval | 0 | 0 | -0.28 | 0.66 | 19.9 | Negative association |
| Self-identified as male | 0 | 0.38 | -0.52 | 0.84 | 35.4 | Negative association |
| Being a cardiologist | 0.49 | 0.45 | 0.49 | 0.77 | 35.6 | Positive association |
