# Supplementary material for: Telemedicine adoption in cardiology: Determinants and predictors identified using Bayesian Model Averaging and Machine Learning
Source: PLOS Digit Health. 2026 Apr 20;5(4):e0001359. doi: 10.1371/journal.pdig.0001359 (PMC13095100; doi:10.1371/journal.pdig.0001359)
Supplement: S3 Table — Top predictors of telemedicine use identified by the XGBoost model in the sensitivity analysis, including mean absolute SHAP values and directionality. (DOCX) [file pdig.0001359.s003.docx]

**S4 Table:** Top predictors of TM Use identified with XGBoost for the sensitivity analysis

| **Feature** | **Mean \|SHAP\| (95%CI)** | **Direction** |
| --- | --- | --- |
| TM knowledge | 1.15 (0.39; 1.99) | Promoting |
| Being a cardiologist | 0.66 (0.22; 1.11) | Promoting |
| TM use suitable for heart failure | 0.56 (0.19; 1.53) | Promoting |
| TM use less suitable in acute events | 0.47 (0.20; 0.95) | Limiting |
| TM use suitable for cardiac arrhythmias | 0.45 (0.12; 1.34) | Promoting |
| Number of average patients per quarter | 0.29 (0.03; 0.85) | Limiting |
| Assessment of the willingness of colleagues to train on TM topic | 0.25 (0.14; 0.41) | Limiting |
| TM use for own health | 0.24 (0.15; 0.34) | Limiting |
| TM use relevant for basic monitoring of ECG | 0.22 (0.11; 0.39) | Promoting |
| Age | 0.19 (0.04; 0.39) | Limiting |
| TM use suitable for monitoring an event | 0.17 (0.05; 0.46) | Promoting |
| TM use relevant for basic monitoring of blood pressure | 0.14 (0.05; 0.21) | Promoting |
| TM use relevant for basic monitoring of weight | 0.14 (0.08; 0.20) | Promoting |
| TM use less suitable for basic monitoring of ECG | 0.13 (0.05; 0.52) | Limiting |
| Self-identified as female | 0.07 (0.02; 0.17) | Promoting |
| TM use less suitable for handling/complications of drugs/medical devices | 0.06 (0.02; 0.10) | Promoting |
| TM use relevant in acute events | 0.05 (0.02; 0.12) | Limiting |
| Willingness to take part in TM training courses | 0.05 (4.2e-03; 0.15) | Limiting |
| TM use less suitable for the consultation of colleagues | 0.05 (0.01; 0.15) | Limiting |
| TM use relevant for handling/complications of drugs/medical devices | 0.03 (0.02; 0.08) | Limiting |
| Willingness to invest for TM | 0.03 (0.01; 0.08) | Promoting |
