# Supplementary material for: Telemedicine adoption in cardiology: Determinants and predictors identified using Bayesian Model Averaging and Machine Learning
Source: PLOS Digit Health. 2026 Apr 20;5(4):e0001359. doi: 10.1371/journal.pdig.0001359 (PMC13095100; doi:10.1371/journal.pdig.0001359)
Supplement: Attachment — Submitted filename: Reviewers_Comments_241025.pdf [file pdig.0001359.s009.pdf]

Rebuttal letter

Dear Mr. Tolentino and the Editorial Team of PLOS Digital Health,

Thank you for your detailed feedback regarding our manuscript PDIG-D-25-00449R2 entitled „Telemedicine adoption in cardiology: determinants and predictors identified using Bayesian model averaging and machine learning.“ We have carefully addressed all editorial requirements and are pleased to resubmit the revised manuscript. Below, we provide a point-by-point response to each of your requests.

Point 1: Competing Interests Statement
We have updated the Competing Interests statement in the online submission system. As no competing interests exist, the statement now reads: „ None declared. “

Point 2: Data Availability Statement
We confirm that the minimal dataset required to replicate all study findings is included in the supporting material of this submission. This includes the survey data underlying all reported analyses. We have updated the Data Availability Statement accordingly to reflect this.

Point 4: Figure Files
All figures have been converted to .tif format using the PLOS NAAS tool and are uploaded as separate files. Each file is within the 10 MB size limit.

Point 5: Supporting Information Legends
A complete list of legends for all Supporting Information files has been added to the manuscript text, immediately before the References section.

We hope that the revised manuscript now fully complies with all journal requirements and that it can proceed to editorial review. We remain grateful for the opportunity to publish in PLOS Digital Health and thank you for your continued support throughout this process.

Sincerely,

Dr. Felix Mühlensiepen
