# Supplementary material for: Telemedicine adoption in cardiology: Determinants and predictors identified using Bayesian Model Averaging and Machine Learning
Source: PLOS Digit Health. 2026 Apr 20;5(4):e0001359. doi: 10.1371/journal.pdig.0001359 (PMC13095100; doi:10.1371/journal.pdig.0001359)
Supplement: Attachment — Submitted filename: Reviewers_Comments_241025.docx [file pdig.0001359.s008.docx]

**Rebuttal letter**

Dear Editor,

Please find attached the revised version of the manuscript number PDIG-D-25-00449 entitled “Predicting telemedicine use among healthcare professionals in cardiology: a Bayesian model averaging and machine learning analysis of German survey data”, which we are re-submitting for publication as an article in the *PLOS Digital Health*.

We would like to warmly thank the reviewers for their comments and suggestions that have been taken carefully into account in reworking this new version of the manuscript. The responses to reviewer and editor questions and all changes made in the manuscript are listed below and written in blue font below to ease the reading. A marked-up version of the manuscript with the changes to the text in red, blue and/or green fonts was made. For better readability we additional added a clear version. All changes made in our manuscript are highlighted in yellow in the herein below responses to reviewers.

Now, we hope that this revised version will be suitable for publication. In all cases, we remain open to any suggestions.

Thank you for considering our work.

Sincerely,

**Point by point response to reviewers**

-----------------------------------------------------------------------------------------------------------------

**Reviewer #1**

The authors incorporated statistical and machine learning approaches to investigate the factors associated with the use of telemedicine using the survey data from 112 healthcare professionals. Univariate analysis was first employed to select the factor most strongly associated with the TM use. This is followed by multivariate analyses, including Bayesian Model Averaging, and Shapley value analysis using the XGBoost. While this study raises an interesting question, its utility, and its additional benefit it brings to the clinical community compared to existing studies, remain unclear. In addition, the methodologies in this manuscript were not described in sufficient details, thus making judging the soundness of their methodologies difficult in its current form. Overall, this manuscript requires substantial improvement before it can be considered for publication.

Major concerns:

1) Essentially, this study employed standard statistical and machine learning approaches to analyze the survey data collected from a previous study from the same group and arrived at a very similar conclusion. Although the authors have provided extensive discussions, the additional benefits this study brings compared to existing studies still need to be further clarified and strengthened. For example, what are the strengths of the methodologies in this study compared to those from the previous studies? Or are there any novel insights that this study provides that were not found by the analytical approaches used in previous studies?

Authors: Thank you for your review and your relevant comments. We value your time and effort. All changes made to the manuscript are highlighted in yellow in our responses to your comments. Regarding the added value of our methodology compared to those from the previous studies, different methods of statistical analysis have been used in the past few decades in order to understand the factors associated with telemedicine (TM) use among users. Most previous investigations of TM adoption among healthcare professionals (HCPs) in cardiology-related fields have employed either qualitative methods (1–3), descriptive statistics (4,5),clustering techniques (21, 36), or logistic regression models (6–8). While these approaches have provided valuable insights, they also carry limitations, particularly in terms of addressing model uncertainty, achieving robust predictive performance, and capturing complex nonlinear relationships between predictors and outcomes.

In this study, we aimed to address these methodological gaps by applying Bayesian model averaging (BMA) alongside modern machine learning (ML) techniques. Unlike conventional regression analyses, which generally identify a single “best-fitting” model and disregard the uncertainty surrounding model specification, BMA formally accounts for this uncertainty by averaging across multiple plausible models weighted by their posterior probabilities (9)This approach yields parameter estimates that are more robust, less sensitive to sampling variability, and therefore more reliable in contexts such as TM adoption, where multiple interdependent factors are at play (10). In parallel, we incorporated ML methods to overcome the limitations of models that assume linear associations between predictors and outcomes. Specifically, we applied the extreme gradient boosting (XGBoost) algorithm, which is widely recognized for its strong predictive accuracy, computational efficiency, and capacity to capture nonlinear and high-dimensional dependencies (11–13).XGBoost also offers built-in mechanisms to address multicollinearity through hierarchical partitioning and regularization, ensuring stability of the results (11–13). These properties allow ML approaches to identify subtle patterns and interactions that traditional models may overlook, thereby enabling more refined insights with direct implications for targeted interventions, policy planning, and resource allocation.

Taken together, the combination of BMA and ML enhances both the inferential rigor and predictive capacity of our analysis. Whereas prior studies have largely confirmed general associations between HCP characteristics and TM adoption, our study adds value by providing stronger evidence regarding the relative importance of predictors, generating more reliable effect estimates, and uncovering nonlinear relationships that yield a more nuanced understanding of adoption dynamics. By moving beyond descriptive or exploratory approaches, our work contributes a more robust and actionable analytical framework for advancing clinical and policy strategies in TM.

To address your comment, we completed the introduction and discussion sections.

Please refer to lines 123-149 in the revised version of the manuscript with track changes

Different statistical analysis approaches have been used in the past few decades in order to understand the factors associated with TM use. Most previous investigations of TM adoption among HCPs in cardiology-related fields have employed either qualitative methods (19–21), descriptive statistics (22,23) clustering techniques (24,25), or logistic regression models (26–28). While these approaches have provided valuable insights, they also carry limitations, particularly in terms of addressing model uncertainty, achieving robust predictive performance, and capturing complex nonlinear relationships between predictors and outcomes.

In this study, we aimed to address these methodological gaps by applying Bayesian model averaging (BMA) alongside modern explainable machine learning (ML) techniques. BMA is designed to identify determinants that are statistically associated with an outcome (e.g., TM use) and provide insights into potential mechanisms and drivers of behavior. In contrast, ML methods such as extreme gradient boosting (XGBoost), prioritize predictive accuracy and are able to capture complex, non-linear relationships that might remain hidden in conventional regression-based approaches. Unlike conventional regression analyses, which generally identify a single “best-fitting” model and disregard the uncertainty surrounding model specification, BMA formally accounts for this uncertainty by averaging across multiple plausible models weighted by their posterior probabilities (29). This approach yields parameter estimates that are more robust, less sensitive to sampling variability, and therefore more reliable in contexts such as TM adoption, where multiple interdependent factors are at play (17) or ML, we applied XGBoost, which is widely recognized for its strong predictive accuracy, computational efficiency, and capacity to capture nonlinear and high-dimensional dependencies (30–32). XGBoost offers built-in mechanisms to address multicollinearity through hierarchical partitioning and regularization, ensuring stability of the results (30–32).

By combining BMA and ML in the present study, we sought to distinguish between explanatory relevance (factors associated with TM use) and predictive relevance (factors that improve classification of TM users vs. non-users). This dual perspective not only provides complementary insights into the determinants of TM adoption among HCPs but also allows us to assess the robustness of findings across distinct analytical frameworks

Please also refer to lines 509-518 in the revised version of the manuscript with track changes (or to lines 509-518 in the revised version without track changes): “This study applied BMA and XGBoost to move beyond confirming general associations by estimating the relative importance of predictors, producing more robust effect estimates, and exploring potential non-linear relationships to provide a more nuanced picture of TM adoption among HCPs. The findings can be summarized as follows. First, across both frameworks, TM knowledge, female sex, and being a cardiologist consistently emerged as core drivers of TM use. Second, the lack of remuneration was identified as a main barrier for TM use in both models. Third, the two approaches also highlighted distinct aspects of TM adoption: BMA emphasized concerns about insufficient patient benefit as a limiting factor, whereas XGBoost identified perceptions of TM suitability for arrhythmias, device monitoring, and blood pressure management as strong predictors. This complementarity underscores the value of combining explanatory and predictive perspectives, explanatory models reveal stable associations that clarify underlying mechanisms, while predictive models capture additional and sometimes non-linear factors that improve classification.”

Please also refer to lines 400-454 in the revised version of the manuscript with track changes:

Most previous investigations of TM adoption among HCPs in cardiology-related fields have relied on qualitative methods (19–21), descriptive statistics (22,23), clustering techniques, or classical logistic regression models (26–28). While these approaches provided valuable insights into general associations, they are limited in addressing model uncertainty, assessing the relative importance of predictors, and capturing complex non-linear relationships. More recently, advanced modelling approaches have been applied to cardiology-related telemedicine data. Koos et al. (2022) used hierarchical logistic regression with cross-validation to disentangle the contributions of patient-, clinician-, and visit-level characteristics to telemedicine use in a large academic cardiovascular center, showing that the individual clinician seen was by far the strongest predictor (AUC=0.83)(33). In contrast, Tisdale et al. (2024) analyzed more than 220,000 patients in the U.S. Veterans Health Administration using multilevel logistic regression and found that 40.5 % of the variability in telehealth use was attributable to the patient level, 30.8 % to the clinician level, and 7 % to the facility level (34). These studies highlight the hierarchical structure of TM adoption but modelled mainly linear effects and did not quantify the relative contribution of individual predictors or explore potential non-linear patterns. Although our survey included only HCPs and could therefore capture only clinician-centered domains, both models consistently identified TM knowledge as the most important factor for TM use. This aligns with prior evidence showing that clinicians’ self-reported technological proficiency, comfort with TM platforms, and positive attitudes toward TM are among the strongest predictors of both current and future TM use, independent of demographic or professional background (35,36) (37)(38)(39).

To the best of our knowledge, no prior work has applied approaches capable of assessing the relative importance of predictors and non-linear relationships to provide a more nuanced understanding of TM adoption dynamics among healthcare professionals in cardiology. Nevertheless, our findings can be robustly situated within the existing literature, as they align with consistently reported determinants of TM use.

Both models in our analysis consistently identified heart failure as a condition perceived to be particularly suitable for TM. This aligns with evidence from clinical trials such as TIM-HF II, which demonstrated the effectiveness of structured telemedical care in heart failure management (33, 34). It is plausible that our respondents considered TM most appropriate in scenarios they were already familiar with, reflecting existing clinical experience rather than openness to entirely new use cases. Interestingly our BMA framework highlighted concerns about insufficient patient benefit as a limiting factor, this likely reflects uncertainty or limited awareness rather than an actual lack of evidence. TM in cardiology has demonstrated significant clinical benefits. A systematic review by Mohammadzadeh et al. found that telecardiology interventions facilitate early diagnosis and treatment, ultimately reducing mortality in patients with cardiovascular disease. Additionally, TM lowers healthcare costs and improves patient quality of life and satisfaction (39). Similarly, studies by Raes et al. and Farabi et al. confirmed that telemonitoring for patients with cardiac implantable electronic devices (CIEDs) and TM integrated with usual care improve clinical outcomes while reducing costs (40, 41). Robust data show that TM improves outcomes, satisfaction, and healthcare utilization in cardiology, particularly in chronic conditions such as heart failure, hypertension, and arrhythmias (14,40,41). These findings suggest that the negative association observed in our model may point to knowledge gaps or misconceptions, underscoring the need for targeted education and awareness initiatives to strengthen confidence in telemedical approaches among healthcare professionals.

Both of our models identified lack of remuneration as the strongest barrier to TM use. This aligns with prior studies showing that unclear or insufficient reimbursement is a key obstacle to TM adoption among clinicians in Germany. Gehrmann et al. (2025) reported that more than half of cardiologists, internists, and general practitioners cited inadequate reimbursement as a major barrier (41) and similar findings were reported by Muehlensiepen et al. (2021) in Rheumatologist (42) and Brauns and Loos (2015)(43). Despite recent reforms, reimbursement for telemedical services remains fragmented and limited (44) and physicians continue to call for remuneration models equivalent to in-person care (45). Our findings thus reinforce the view that structural financial barriers remain a major bottleneck for sustainable TM implementation for German HCPs.”

2) Overall, the methodology and the analyses in this study need to be described in much more specific details in order to properly assess the validity of this study, as various crucial information seems to be missing, and some of the descriptions are also unclear and confusing. My specific concerns about these descriptions will be detailed in the following comments, but here are some of the most relevant concerns:

Authors: To address your comment on the methodology and analysis, we expanded the statistical analysis part in the methods section.

Please refer to lines 509-518 in the revised version of the manuscript with track changes (or to lines 509-518 in the revised version without track changes): “All statistical analyses were performed using R software 4.5.0® (R Core Team, Vienna, Austria) for Windows 11©.

*Data management*

Independent variables were defined based on all columns except the outcome variable, TM use. Ordered variables were retained as ordered factors, and nominal categorical variables with more than two levels were one-hot encoded, with one reference category omitted per variable to prevent multicollinearity (21). Missing values were handled as a separate category, under the assumption that nonresponse may reflect systematic differences in TM use. After these steps, the dataset included 53 independent covariates.

*Bayesian model averaging (BMA) analysis*

We implemented a structured three-step pipeline to identify determinants of TM use. First, collinearity screening was conducted by examining pairwise correlations and calculating the variance inflation factor (VIF), with variables exhibiting VIF > 2.5 excluded to mitigate multicollinearity (24) . Next, LASSO regression was applied to reduce dimensionality and retain the most informative variables. Optimal penalization parameters were identified using five-fold cross-validation (CV), and variables with non-zero coefficients at the optimal lambda were retained. These variables were subsequently entered into a BMA framework with logistic regression. BMA averages across all candidate models, weighting by posterior probabilities, thus accounting for model uncertainty (Depaoli et al. 2021). This process provided posterior inclusion probabilities (PIPs) for each feature, indicating the likelihood that the variable has a non-zero effect on TM use. Posterior mean coefficients and standard deviations were calculated across all models (full BMA estimates), and we also summarized results from the single highest posterior probability model and the five highest posterior probability models for comparison. Variables with non-zero PIPs were considered determinants of TM use, with higher PIPs reflecting stronger evidence of association. Visual summaries of group-level differences were produced using radar plots, where continuous and ordinal variables were normalized to a 0–1 range using min–max scaling and summarized by group medians, whereas dichotomous variables were summarized by group proportions. BMA was performed using the BMA package (version 3.18.17) (27). Regarding priors for BMA, we assumed that all candidate models were equally likely a priori (same prior weight).

*Machine learning analysis*

To complement the BMA analysis and leverage the predictive capacity of ML, we applied a binary XGBoost model. Unlike regression models, XGBoost handles multicollinearity through its tree-based structure, so no pre-filtering for collinearity was performed (30–32) To avoid biased evaluation, a nested CV framework was applied. The dataset was first split into a 70% training set and a 30% holdout test set using stratified sampling to preserve the proportion of TM users and non-users. Within the training set, a 10-fold outer CV was performed, with each fold sequentially serving as validation while the remaining folds formed the training subset. Within each outer training fold, a 10-fold inner CV was conducted to tune hyperparameters. Due to computational constraints, 30 random hyperparameter combinations were tested for each fold, with learning rate ([0.05-1]), maximum depth ([3-7]), minimum sum of instance weights (hessian) needed in a child node ([1-10]), subsample ([0.5-1]), and subsample ratio of columns when constructing each tree ([0.5-1]) varied within pre-specified ranges. The scale_pos_weight parameter was set to the ratio of majority to minority class to account for class imbalance. Hyperparameters were optimized by maximizing the mean area under the receiver operating characteristic curve (AUROC) across inner folds. The best hyperparameter configuration for each outer fold was used to train an XGBoost model, which was then evaluated on the corresponding outer validation set.

Following nested CV, the hyperparameter configuration yielding the highest AUROC across outer folds was selected to train a final XGBoost model on the full training set. This model was evaluated on the independent 30% holdout test set. Evaluation metrics included AUROC with 95% confidence intervals, accuracy with 95% confidence intervals, F1-score, and additional metrics such as precision, recall, and specificity. The F1 score is a measure of predictive performance, combining precision and recall into a single value, ranging from 0 (poor performance) to 1 (perfect performance).

The XGBoost algorithm was computed using the xgboost package version 1.7.8.1 (50). The caret package version 7.0-1 was used to calculate the confusion matrix (51). The pROC package version 1.18.5 (52) was used to calculate the AUROC, while the MLmetrics package version 1.1.3 (53) was used to compute evaluation performance metrics not provided by the caret package. The stratified sampling was performed with the splitTools package version 1.0.1 (54) . Model training, cross-validation, and hyperparameter tuning were carried out with the mlexperiments package (55) in combination with the mllrnrs package (56), which provide standardized machine learning pipelines and learner interfaces.

To enhance the interpretability of the XGBoost model, SHapley Additive exPlanations (SHAP) values were calculated using the holdout test set to quantify the contribution of each predictor to the model's output. SHAP values reflect the individual contribution of each predictor by evaluating its marginal impact on the model’s output (57,58).Predictors that directed the model toward TM use were considered promoting factors, whereas predictors directing the model toward non-use were considered limiting factors. Directionality was inferred using a hybrid heuristic. For binary and sparse features, mean SHAP values were compared across groups, while for continuous features, generalized additive models (GAMs) were fitted to SHAP values, and the average derivative of the fitted curve was used to determine association direction. When GAMs failed to converge, Spearman’s rank correlation between the feature and its SHAP values served as a fallback.

*Sensitivity analysis*

A sensitivity analysis was performed by excluding individuals with missing values to evaluate the robustness of our findings.”

In addition, all scripts used for analysis have been made publicly available, as detailed in the Data Availability Statement: “The data (participant data, data dictionary, and other related documents) that support the findings of this study are not publicly available but can be available from the corresponding author upon reasonable request. The codes used in the analysis of this study is publicly available at: https://doi.org/10.5281/zenodo.17047856.”.

a) To properly evaluate a machine learning model, the cohort used for reporting the performance of the predictive models should never be used during the variable selection and the model training. While the author used cross-validation in the training process, it is unclear whether the variable selection via univariate analysis was conducted using the entire cohort, or within the training cohort of each fold. If it is the former case, the testing partition would have been inadvertently used during the variable selection stage, which would result in information leakage and thereby inflate the performance metrics. Please clarify the independence of data partition in each stage of the analysis. If there is potential information leakage, the authors could either conduct variable selection only within the training data of each fold or use an independent evaluation cohort for assessing model performance.

Authors: Regarding your concern about potential information leakage during variable selection, we would like to clarify the distinction between the Bayesian Model Averaging (BMA) and machine learning (ML) analyses in our study. For the BMA analysis, variable selection was indeed conducted on the full dataset prior to model fitting. However, BMA was used as an inferential framework rather than a predictive model. In this context, preselecting variables based on the entire cohort is consistent with standard practice in regression modeling, where the goal is to estimate the posterior inclusion probabilities of predictors rather than to generate out-of-sample predictions. To improve clarity, we have revised the description of the BMA feature selection process in the Methods section. We also updated the way the feature selection was performed.

For the ML models, no variable selection was performed using the entire dataset. All candidate predictors, excluding the outcome and individual identifiers, were pre-specified before any cross-validation (CV). The XGBoost algorithm internally handles feature selection and assigns importance through hierarchical splitting and regularization. Importantly, hyperparameter tuning and model evaluation were conducted using nested CV, ensuring that the holdout fold in each outer iteration remained completely independent from both training and hyperparameter selection. This approach prevents information leakage and preserves the validity of performance estimates. By relying on the model’s internal mechanism to identify important features rather than performing preselection on the full dataset, we maintained proper data independence across all stages of ML training and evaluation, which is consistent with best practices in predictive modeling.

Please refer to lines 509-518 in the revised version of the manuscript with track changes (or to lines 509-518 in the revised version without track changes): “*Data management*

Independent variables were defined based on all columns except the outcome variable, TM use. Ordered variables were retained as ordered factors, and nominal categorical variables with more than two levels were one-hot encoded, with one reference category omitted per variable to prevent multicollinearity (21). Missing values were handled as a separate category, under the assumption that nonresponse may reflect systematic differences in TM use. After these steps, the dataset included 53 independent covariates.

*Bayesian model averaging (BMA) analysis*

We implemented a structured three-step pipeline to identify determinants of TM use. First, collinearity screening was conducted by examining pairwise correlations and calculating the variance inflation factor (VIF), with variables exhibiting VIF > 2.5 excluded to mitigate multicollinearity (24) . Next, LASSO regression was applied to reduce dimensionality and retain the most informative variables. Optimal penalization parameters were identified using five-fold cross-validation (CV), and variables with non-zero coefficients at the optimal lambda were retained. These variables were subsequently entered into a BMA framework with logistic regression. BMA averages across all candidate models, weighting by posterior probabilities, thus accounting for model uncertainty (Depaoli et al. 2021). This process provided posterior inclusion probabilities (PIPs) for each feature, indicating the likelihood that the variable has a non-zero effect on TM use. Posterior mean coefficients and standard deviations were calculated across all models (full BMA estimates), and we also summarized results from the single highest posterior probability model and the five highest posterior probability models for comparison. Variables with non-zero PIPs were considered determinants of TM use, with higher PIPs reflecting stronger evidence of association. Visual summaries of group-level differences were produced using radar plots, where continuous and ordinal variables were normalized to a 0–1 range using min–max scaling and summarized by group medians, whereas dichotomous variables were summarized by group proportions. BMA was performed using the BMA package (version 3.18.17) (27). Regarding priors for BMA, we assumed that all candidate models were equally likely a priori (same prior weight).

*Machine learning analysis*

To complement the BMA analysis and leverage the predictive capacity of ML, we applied a binary XGBoost model. Unlike regression models, XGBoost handles multicollinearity through its tree-based structure, so no pre-filtering for collinearity was performed (30–32)
b) The 34 items of the questionnaire, which is the central part of the study design, should be provided in the manuscript. In addition to providing the complete set of questionnaire items, how the items related to TM barriers were defined requires further clarification. According to the survey questions in reference 19, the items related to the TM barriers are under the question “What prevents you from using telemedicine? (Multiple selections were possible.)“ Does answering this question directly indicate that the participant did not use telemedicine? If so, the link between TM use and these barrier-related variables should be self-evident. In addition, there are some barrier-related variables that somehow “promote” the use of TM for these participants (according to Table 4). If these barrier-related variables reflect the participating clinicians’ personal use of TM rather than their opinion on the TM use as a public agenda (as indicated by reference 19), this finding will be very counterintuitive and contradicts the authors’ own conclusions in the Discussion section that “TM remains underutilized due to inadequate reimbursement.”

Authors: We thank the reviewer for this valuable comment and the opportunity to clarify how the questionnaire items related to telemedicine (TM) barriers were operationalized.

The questionnaire included 34 items in total; the central section focused on barriers to TM use and was based on the question: “What prevents you from using telemedicine?” (multiple selections possible). Each listed barrier (e.g., data security concerns, lack of reimbursement, insufficient technical infrastructure) was used to generate a dichotomous variable, coded as 1 if the participant selected the respective barrier and 0 otherwise. Thus, the original multi-response item was transformed into a set of binary variables using one-hot encoding.

Participants who answered this question were not exclusively non-users of telemedicine. Rather, respondents were asked to indicate which aspects they perceived as barriers to TM use, irrespective of their actual level of usage. Consequently, some clinicians who reported specific barriers (e.g., data security concerns) may still have used TM in their practice, whereas others may not have. This approach allowed us to capture both perceived and experienced barriers among the full sample, including TM users and non-users.

We acknowledge that this may have resulted in certain barrier-related variables showing positive associations with TM use in the multivariate models. This likely reflects that some active users are more aware of specific limitations or systemic shortcomings of TM (e.g., reimbursement issues) rather than these barriers “promoting” TM use. We have clarified this distinction in the revised manuscript and added the full list of questionnaire items in the Supplementary Material.

Specific comments:

1) In the Methods section, Variable Selection subsection:

a) “One-hot encoding was applied to all categorical variables with more than two categories.” Judging from the results presented by the authors, ordinal variables were also treated as categorical variables, which limited the explainability of the results. For example, only the age group between 40 and 49 years was considered relevant to predicting TM use, whereas how younger and older age groups are associated with TM use remained unknown. Using approaches that properly handle ordinal variables would greatly improve the explainability of their findings.

Authors: We agree that treating ordinal variables as nominal could limit interpretability. In the revised analysis, ordinal variables were explicitly retained as ordered factors without contrast coding, preserving their natural ranking. Only nominal categorical variables with more than two categories were subjected to one-hot encoding, with one reference category omitted per variable to prevent multicollinearity. This approach allows the model to appropriately reflect trends across ordered levels, such as age groups, thereby enhancing the interpretability of the results.

Please refer to lines 509-518 in the revised version of the manuscript with track changes (or to lines 509-518 in the revised version without track changes): “Ordered variables were retained as ordered factors, and nominal categorical variables with more than two levels were one-hot encoded, with one reference category omitted per variable to prevent multicollinearity.”

b) As mentioned in the Major Concerns, the cohort used for reporting the final model performance (Table 2) should not be used during variable selection and the model training. Please clarify if this is the case in the manuscript.

Authors: We confirm that the cohort used to report the final model performance was not used during variable selection or model training. The dataset was first partitioned into a 70% training set and a 30% holdout test set using stratified sampling to preserve the proportion of TM users and non-users. All model training and hyperparameter tuning were performed exclusively within the training set. Specifically, a 10-fold outer cross-validation (CV) was conducted, where each fold sequentially served as a validation subset while the remaining folds formed the training subset. Within each outer training fold, a 10-fold inner CV was performed for hyperparameter optimization. Hyperparameters were selected by maximizing the mean area under the receiver operating characteristic curve (AUROC) across inner folds. The best hyperparameter set from each outer fold was used to train a fold-specific XGBoost model, which was evaluated on the corresponding outer validation set. Finally, the hyperparameter configuration achieving the highest AUROC across all outer folds was used to train a final XGBoost model on the full training set, and its performance was assessed solely on the independent 30% holdout test set.

Please refer to lines 509-518 in the revised version of the manuscript with track changes (or to lines 509-518 in the revised version without track changes): “The dataset was first split into a 70% training set and a 30% holdout test set using stratified sampling to preserve the proportion of TM users and non-users. Within the training set, a 10-fold outer CV was performed, with each fold sequentially serving as validation while the remaining folds formed the training subset. Within each outer training fold, a 10-fold inner CV was conducted to tune hyperparameters. Due to computational constraints, 30 random hyperparameter combinations were tested for each fold, with learning rate ([0.05-1]), maximum depth ([3-7]), minimum sum of instance weights (hessian) needed in a child node ([1-10]), subsample ([0.5-1]), and subsample ratio of columns when constructing each tree ([0.5-1]) varied within pre-specified ranges. The scale_pos_weight parameter was set to the ratio of majority to minority class to account for class imbalance. Hyperparameters were optimized by maximizing the mean area under the receiver operating characteristic curve (AUROC) across inner folds. The best hyperparameter configuration for each outer fold was used to train an XGBoost model, which was then evaluated on the corresponding outer validation set.

Following nested CV, the hyperparameter configuration yielding the highest AUROC across outer folds was selected to train a final XGBoost model on the full training set. This model was evaluated on the independent 30% holdout test set.”

2) In the Methods section, Machine learning analysis:

a) On “A balanced 70%/30% train/test split was created using downsampling, ensuring no HCPs appeared in both training and test datasets.”: Please be more specific about how the downsampling was performed.

Authors: We thank you for pointing out this inaccuracy. The term “downsampling” was incorrectly used in the original manuscript. In fact, the dataset was split using stratified random sampling to ensure that the proportion of TM users and non-users was preserved in both the training and test sets. No healthcare professionals (HCPs) appeared in both datasets, maintaining independence between the training and holdout sets. This correction has been implemented in the revised manuscript.

b) By creating a “balanced” training/testing split, did the author intend to convey that the number of HCPs who use TM equals those who do not use TM?

Authors: We thank you for this comment and acknowledge the potential for misunderstanding. The term “balanced” was not intended to imply that the number of TM users and non-users was made equal. Rather, stratified sampling was applied to maintain the original proportion of TM users and non-users in both the training and holdout test sets, avoiding bias in model evaluation while preserving class distributions. To clarify this, we have revised the manuscript accordingly.

Please refer to lines 509-518 in the revised version of the manuscript with track changes (or to lines 509-518 in the revised version without track changes): “To avoid biased evaluation, a nested CV framework was applied. The dataset was first split into a 70% training set and a 30% holdout test set using stratified sampling to preserve the proportion of TM users and non-users. Within the training set, a 10-fold outer CV was performed, with each fold sequentially serving as validation while the remaining folds formed the training subset. Within each outer training fold, a 10-fold inner CV was conducted to tune hyperparameters. Due to computational constraints, 30 random hyperparameter combinations were tested for each fold, with learning rate ([0.05-1]), maximum depth ([3-7]), minimum sum of instance weights (hessian) needed in a child node ([1-10]), subsample ([0.5-1]), and subsample ratio of columns when constructing each tree ([0.5-1]) varied within pre-specified ranges. The scale_pos_weight parameter was set to the ratio of majority to minority class to account for class imbalance. Hyperparameters were optimized by maximizing the mean area under the receiver operating characteristic curve (AUROC) across inner folds. The best hyperparameter configuration for each outer fold was used to train an XGBoost model, which was then evaluated on the corresponding outer validation set.”

c) Was the downsampling conducted completely at random? In addition, was the random downsampling performed independently for each fold of the cross-validation? Or done only once prior to cross-validation?

Authors: We thank you for raising this point. The term “downsampling” in the original manuscript was incorrect; instead, we applied stratified random sampling. The dataset was split once prior to cross-validation, with 70% assigned to the training set and 30% to the holdout test set, while maintaining the original proportion of TM users and non-users in both subsets. Cross-validation folds within the training set were then created using stratified sampling, ensuring that each fold preserved the class distribution. No additional random sampling or downsampling was performed independently for each fold. This approach ensures that performance evaluation is unbiased while preserving the original class distribution.

Please refer to lines 509-518 in the revised version of the manuscript with track changes (or to lines 509-518 in the revised version without track changes): “To avoid biased evaluation, a nested CV framework was applied. The dataset was first split into a 70% training set and a 30% holdout test set using stratified sampling to preserve the proportion of TM users and non-users. Within the training set, a 10-fold outer CV was performed, with each fold sequentially serving as validation while the remaining folds formed the training subset. Within each outer training fold, a 10-fold inner CV was conducted to tune hyperparameters. Due to computational constraints, 30 random hyperparameter combinations were tested for each fold, with learning rate ([0.05-1]), maximum depth ([3-7]), minimum sum of instance weights (hessian) needed in a child node ([1-10]), subsample ([0.5-1]), and subsample ratio of columns when constructing each tree ([0.5-1]) varied within pre-specified ranges. The scale_pos_weight parameter was set to the ratio of majority to minority class to account for class imbalance. Hyperparameters were optimized by maximizing the mean area under the receiver operating characteristic curve (AUROC) across inner folds. The best hyperparameter configuration for each outer fold was used to train an XGBoost model, which was then evaluated on the corresponding outer validation set.”

d) Was the testing set also downsampled? If so, it may inadvertently introduce unwanted selection bias, and the reported values for positive/negative will no longer be valid since it alters the data distribution

Authors: The holdout test set was not subjected to downsampling or any other form of resampling. It retained the original distribution of TM users and non-users in the cohort. Consequently, performance metrics reported on this set, including AUROC, accuracy, and F1-score, accurately reflect the model’s predictive ability without introducing selection bias.

e) “ensuring no HCPs appeared in both training and test datasets” seems to suggest that there are cases where an HCP has filled multiple questionnaires. Please specify if this is true and justify the use of multiple questionnaires per HCP if needed.

Authors: Thank you for raising this point. We clarify that each healthcare professional completed only a single questionnaire, and every entry in the dataset corresponds to a unique respondent. Consequently, no individual appears in both the training and test datasets, ensuring independence between these subsets. The manuscript text has been updated to explicitly reflect this.

Please refer to lines 509-518 in the revised version of the manuscript with track changes (or to lines 509-518 in the revised version without track changes): “The dataset was first split into a 70% training set and a 30% holdout test set using stratified sampling to preserve the proportion of TM users and non-users.”

f) It was suggested in the abstract that nested cross-validation was used. However, how such nested cross-validation was conducted was either not described in sufficient detail or was described inaccurately. A truly nested cross-validation would consist of an outer loop and an inner loop of the cross-validation. Nevertheless, there is no clear description of how such nested cross-validation was conducted. Additionally, regarding “Final tuning hyperparameter values were chosen based on the best out-of-fold AUROC. Model performance was assessed using AUROC and F1 scores.” Please confirm if the data partition used for selecting the hyperparameter is different from the partition used for assessing the final model performance.

Authors: To clarify, we applied a truly nested cross-validation (CV) framework to ensure unbiased evaluation. The dataset was first split into a 70% training set and a 30% holdout test set using stratified sampling to preserve the proportion of TM users and non-users. Within the training set, a 10-fold outer CV was conducted, with each fold sequentially serving as a validation set while the remaining folds formed the training subset. Within each outer training fold, a 10-fold inner CV was performed specifically for hyperparameter tuning. Hyperparameters were optimized by maximizing the mean area under the receiver operating characteristic curve (AUROC) across the inner folds. The best hyperparameter configuration for each outer fold was then used to train an XGBoost model, which was evaluated on the corresponding outer validation set.

We also confirm that the holdout test set used for final model performance assessment was completely independent of the training data and was not used during hyperparameter selection, ensuring that performance metrics, including AUROC and F1 scores, reflect unbiased evaluation.

Please refer to lines 509-518 in the revised version of the manuscript with track changes (or to lines 509-518 in the revised version without track changes): “To avoid biased evaluation, a nested CV framework was applied. The dataset was first split into a 70% training set and a 30% holdout test set using stratified sampling to preserve the proportion of TM users and non-users. Within the training set, a 10-fold outer CV was performed, with each fold sequentially serving as validation while the remaining folds formed the training subset. Within each outer training fold, a 10-fold inner CV was conducted to tune hyperparameters. Due to computational constraints, 30 random hyperparameter combinations were tested for each fold, with learning rate ([0.05-1]), maximum depth ([3-7]), minimum sum of instance weights (hessian) needed in a child node ([1-10]), subsample ([0.5-1]), and subsample ratio of columns when constructing each tree ([0.5-1]) varied within pre-specified ranges. The scale_pos_weight parameter was set to the ratio of majority to minority class to account for class imbalance. Hyperparameters were optimized by maximizing the mean area under the receiver operating characteristic curve (AUROC) across inner folds. The best hyperparameter configuration for each outer fold was used to train an XGBoost model, which was then evaluated on the corresponding outer validation set.”

g) Since there are multiple XGBoost models from the 10-fold cross-validation, please clarify how the Shapley values were combined and presented.

Authors: To clarify, although multiple XGBoost models were trained during the 10-fold cross-validation process, SHapley Additive exPlanations (SHAP) values were calculated only on the independent holdout test set using the final XGBoost model trained on the full training data with the best-performing hyperparameters. This approach ensures that the SHAP values reflect the contribution of each predictor to the final model's output without introducing bias from fold-specific models.

Please refer to lines 509-518 in the revised version of the manuscript with track changes (or to lines 509-518 in the revised version without track changes): “To enhance the interpretability of the XGBoost model, SHapley Additive exPlanations (SHAP) values were calculated using the holdout test set to quantify the contribution of each predictor to the model's output.”.

3) In the Results section:

a) The purpose of each analysis was not clearly stated in the main text, and the writing should be improved to provide a coherent flow of logic.

Authors: To address your comment, we have modified the entire results section. Please refer to lines 509-518 in the revised version of the manuscript with track changes (or to lines 509-518 in the revised version without track changes): “*BMA analysis*

Of the 53 independent covariates initially considered, 32 (60%) were retained after excluding collinear variables. Among these 32, 12 (38%) were selected using Least Absolute Shrinkage and Selection Operator (LASSO) regression and subsequently included in the BMA analysis. Examination of posterior model probabilities revealed model uncertainty. No single model dominated, with the highest posterior model accounting for only 11.0% of the total weight, and the top five models together representing 41.3%. Estimates based on only the top model, or approximated across the top five models, differed from the fully model-averaged estimates (Table 1, Figure 1). In contrast, posterior means from the full BMA, which incorporate information from the entire model space, provided more stable estimates that were often shrunk toward zero.

**Figure 1**: Coefficient estimates for each predictor variable under three modeling perspectives: the highest posterior probability single model (“Top model”), an approximation averaged over the top five models (“Top 5 models”), and the fully Bayesian model-averaged estimates (“BMA”). The points represent the estimated coefficient effect sizes, while the error bars show the posterior standard deviations derived from the full BMA analysis, reflecting uncertainty in these averaged estimates. The “Top model” and “Top 5 models” estimates do not include uncertainty intervals because these approximations do not capture model-averaged uncertainty. The full “BMA” estimates incorporate all plausible models weighted by their posterior probabilities, shrinking coefficients toward zero when evidence for inclusion is weak and properly reflecting parameter uncertainty.

A total of 12 determinants of TM use were identified (Table 1, Figure 2). Positive associations were found for TM knowledge, being a cardiologist, self-identifying as female, perceiving TM as suitable for heart failure and for monitoring an event, considering absence of remuneration and data security as barriers to TM, and perceiving TM as relevant for extending the aftercare interval and for basic ECG monitoring. Negative associations were observed for considering insufficient data on patient benefit as a TM barrier, and for perceiving TM as less suitable in acute events or for extending the aftercare interval (Table 1).

The sensitivity analysis produced similar findings, identifying eight determinants, all of which overlapped with the main analysis and showed consistent directions of association (Figure 2, Table S1, Figure S1). However, in this analysis, data security, the relevance of TM for ECG monitoring and aftercare extension, and the unsuitability of TM in acute events were not retained as determinants.

**Table 1**: Factors Identified Through BMA and Their Association With TM Use

The determinants identified through BMA were used to describe the profiles of HCPs in cardiology who use and do not use TM (Figure 2). Profiles are presented for both the main and sensitivity analyses. The percentages correspond to the proportion of HCPs endorsing each specified response. For example, 100% (the outer line of the radar chart) indicates that all HCPs selected the given response (e.g., self-identifying as female), whereas 0% (the inner line) indicates that none selected it.

**Figure 2**: Profile of healthcare professionals (HCPs) in cardiology using telemedicine (TM) versus non-users. The radar plot compares key determinants, with TM users (green) and non-users (red).

*Machine Learning analysis*

The XGBoost model demonstrated strong performance, with an AUROC of 0.88 (95% CI: 0.75–1.00), an accuracy of 0.79 (95% CI: 0.62–0.91), a sensitivity of 79%, a specificity of 80%, and an F1 score of 0.81 (Table 2).

**Table 2**: Performance of the final XGBoost model

***Note***: The purpose of the McNemar’s test is to test whether the number of false positives and false negatives are statistically equal.

Several of the most influential predictors identified by SHAP analysis (e.g., TM knowledge, being a cardiologist, and female sex) were also among the key determinants highlighted in the BMA analysis. Figure 3, Table 3).

The ML framework identified additional predictors that did not emerge as determinants in the BMA models. For example, perceptions of TM suitability for arrhythmias, pacemaker and defibrillator therapy, and for monitoring blood pressure ranked highly in predictive importance, reflecting the model’s ability to capture more complex or non-linear associations. Conversely, factors such as concerns about insufficient patient benefit, which were negatively associated with TM use in BMA, did not contribute substantially to the predictive performance of the ML model.

In terms of directionality, SHAP values indicated that several factors promoted TM use, including TM knowledge, being a cardiologist, female sex, perceiving TM as suitable for arrhythmias and heart failure, higher patient volume per quarter, and viewing TM as relevant for managing complications of drugs and medical devices. In contrast, limiting factors included perceiving TM as unsuitable for acute events or aftercare extension, older age, using TM for own health, and concerns about implementation effort (Table 3, Figure 3).

The sensitivity analysis yielded broadly consistent findings, although some predictors identified in the main model were not retained, such as perceptions of insufficient remuneration as a barrier or suitability for hypertension (Table 2, Figure S2, Table S2). At the same time, new predictors emerged, such as the perception that TM is less suitable for basic ECG monitoring or for consulting colleagues. Certain predictors shifted directionality. For instance, the number of patients per quarter and willingness to invest in TM showed opposite associations compared with the main model. These discrepancies emphasize that while the overall predictive structure of the ML model was stable, the importance of individual features may vary depending on the population considered.

**Figure 3**: Feature importance according to SHAP values for the prediction of TM use.

SHAP value (x-axis) for each HCP and feature are represented with a point. Positive SHAP values imply an impact to the model toward wanting TM use, while negative values impact the model toward not using TM. For each feature, the mean and 95% CI of the absolute SHAP values are reported on the right of the graph. High SHAP value (in absolute value) indicate a high impact on the model output. Red colors indicate that an HCP either answered yes to the considered question (y-axis) regarding categorical feature or tend toward high values for continuous features (e.g., age), while blue colors refer either to “no” answers for categorical features or low values for continuous features, and grey colors refer to missing answers.

**Table 3**: Top predictors of TM Use identified with XGBoost

*BMA and XGBoost comparison*

The explanatory and predictive frameworks converged on several key factors while also highlighting different aspects of TM adoption (Table 4). Both BMA and XGBoost identified TM knowledge, female sex, and being a cardiologist as robust determinants of TM use. These variables demonstrated both a consistent statistical association and a strong contribution to predictive performance, suggesting that they represent core drivers of adoption in cardiology practice. Similarly, perceptions that TM is unsuitable for acute events or for extending aftercare intervals were negatively associated with use in BMA and also reduced predictive probability in XGBoost, reinforcing their importance as barriers.

Beyond these points of convergence, the two approaches diverged in meaningful ways. BMA, by design, emphasized parsimonious associations with stable posterior inclusion probabilities, highlighting concerns about insufficient patient benefit as a limiting factor, an effect that did not substantially improve the predictive capacity of the XGBoost model. Conversely, XGBoost identified perceptions of TM suitability for arrhythmias, device monitoring, and blood pressure management as strong predictors, even though these factors did not consistently emerge as explanatory determinants in BMA. These differences underscore the complementary nature of the two methods. BMA provides insights into determinants with stable associations, while ML captures a broader set of variables that enhance classification accuracy, including those with weaker or more context-dependent effects.

Importantly, one feature (perceptions of TM suitability for event monitoring) exhibited incongruent behavior across approaches, showing a positive association in one framework (BMA) but a limiting effect in the other (XGBoost). Such discrepancies highlight the value of applying both explanatory and predictive methodologies, as they draw attention to variables whose influence on TM adoption may depend on context, measurement, or modeling assumptions.

**Table 4**: Comparison of the feature impact on TM use depending on the statistical method use

***Note***: empty cells refer to features that were not selected in the models.”.

b) On the subsection title, “Patient characteristics”: the participants in this study are not patients but healthcare professionals instead.

Authors: Thank you for noticing. We corrected this mistake.

Please refer to lines 509-518 in the revised version of the manuscript with track changes (or to lines 509-518 in the revised version without track changes): “Healthcare professional characteristics”.

c) Methods such as BMA and XGBoost should be cited upon their first occurrence.

Authors: We thank the reviewer for this helpful comment. We have added the appropriate references for BMA and XGBoost at their first mention in the Methods section.

d) Table 2: The purpose of McNemar's test was not described in the manuscript.

Authors: The purpose of the McNemar’s test is to test whether the number of false positives and false negatives are statistically equal. We have included its description in the manuscript.

Please refer to lines 509-518 in the revised version of the manuscript with track changes (or to lines 509-518 in the revised version without track changes): “The purpose of the McNemar’s test is to test whether the number of false positives and false negatives are statistically equal”.

e) Some of the findings also seem to be self-contradictory. Most notably, both “TM use relevant in acute events” and “TM use less suitable in acute events” were considered as limiting factors in Table 3.

Authors: The two items referred to—“TM use relevant in acute events” and “TM use less suitable in acute events”—originate from two distinct sections of the questionnaire addressing different conceptual dimensions of TM use.

The first item (“TM use relevant in acute events”) was part of a question asking respondents to indicate contexts in which they considered TM to be particularly useful (e.g., transmission of an ECG in a suspected myocardial infarction or remote triage in emergency care).

The second item (“TM use less suitable in acute events”) was part of a separate question focusing on limitations and perceived barriers to TM use in various clinical scenarios.

The coexistence of both items does not reflect contradictory wording in the survey but rather acknowledges that clinicians may perceive specific subtypes of acute situations as appropriate for TM (e.g., diagnostic support or initial triage) while regarding other acute scenarios as less suitable (e.g., those requiring direct physical intervention or complex multidisciplinary decision-making).

We have revised the manuscript to make this distinction explicit and to clarify that the two items stem from different sections of the questionnaire capturing perceived potentials versus limitations of TM in acute care contexts.

f) Table 4 shows a marked divergence between the findings of the two analytical approaches. Although the authors partially addressed this by focusing solely on the congruent findings across these two approaches, it would be more methodologically sound to conduct dedicated sensitivity analyses to investigate how much the findings vary between different initialization criteria or different folds of the cross-validation.

Authors: We thank the reviewer for this suggestion. We would like to clarify that BMA and XGBoost are distinct analytical frameworks with fundamentally different underlying mechanisms. BMA, as implemented with the bic.glm() function in R, estimates posterior model probabilities using the Bayesian Information Criterion (BIC), thereby averaging over a large set of plausible models rather than relying on a single specification. In contrast, XGBoost is a gradient boosting ensemble method, where variability arises from the resampling and boosting process.

In our study, BMA results reflect posterior inclusion probabilities derived from the full model space considered by bic.glm(), which mitigates the risk of overstating evidence based on a single model. XGBoost was trained using a 10-fold nested cross-validation framework with stratified sampling, which inherently accounts for variability due to fold assignment and class imbalance. The top predictors and performance metrics reported reflect estimates obtained across the entire nested CV process.

Although additional sensitivity analyses could be conducted using alternative random seeds or fold assignments, we emphasize that our current methodology, averaging across the full model space for BMA and stratified nested CV for XGBoost, provides a robust and reliable assessment of predictor importance and model performance. These procedures follow standard practice for each method and ensure that the reported findings are not dependent on a single model specification or a single partition.

4) In the Discussion section, Strengths and Limitations subsection:

a) “advanced statistical methodologies” is an overstatement, since BMA has been used for decades.

Authors: We appreciate the reviewer’s point and agree that BMA is a well-established approach. To avoid overstating novelty, we have revised the wording to “established sophisticated statistical methodologies.” This phrasing acknowledges the long-standing use of BMA, while distinguishing it from more basic statistical tests. Please refer to lines 509-518 in the revised version of the manuscript with track changes (or to lines 509-518 in the revised version without track changes).

b) “The five models with the highest posterior probabilities (CPPs) together accounted for only 24.1% of the total model weight…This reinforces the importance of our BMA approach, which averages across the full set of plausible models, thereby providing effect estimates that are more robust and less sensitive to arbitrary variable inclusion decisions.” A low CPP when using the top-five models does not automatically lead to the conclusion that BMA can provide more robust estimates when using the full set of plausible models. Quantitative evidence needs to be provided.

Authors: You are correct that a low cumulative posterior probability (CPP) among the top models does not, on its own, justify the robustness of BMA estimates. To address this, we now provide quantitative evidence.

Please refer to lines 509-518 in the revised version of the manuscript with track changes (or to lines 509-518 in the revised version without track changes): “Examination of posterior model probabilities revealed model uncertainty. No single model dominated, with the highest posterior model accounting for only 11.0% of the total weight, and the top five models together representing 41.3%. Estimates based on only the top model, or approximated across the top five models, differed from the fully model-averaged estimates (Table 1, Figure 1). In contrast, posterior means from the full BMA, which incorporate information from the entire model space, provided more stable estimates that were often shrunk toward zero.”.

Please also refer to Table 1 and Figure 1.

Please also refer to lines 509-518 in the revised version of the manuscript with track changes (or to lines 509-518 in the revised version without track changes): “In our analysis, the five models with the highest posterior probabilities together accounted for only 41.3% of the total model weight, indicating considerable model uncertainty. Many variables displayed larger effect sizes in the “top model” or across the “top five models” compared with the full BMA estimates, illustrating how reliance on a small subset of models can exaggerate associations by neglecting the broader model space. Because the posterior probabilities of the leading models were relatively low, coefficients derived from them, essentially maximum likelihood estimates conditional on a single specification, risk overstating evidence. In contrast, the BMA posterior means integrate information across the entire space of plausible models, shrinking coefficients toward zero when support for a predictor is weak and thus yielding more stable and reliable estimates. These findings underscore that restricting inference to a single “best” model, or even to a handful of high-probability models, would disregard most of the available information. By averaging over the complete model space, BMA provides more robust inference and guards against spurious conclusions driven by arbitrary model selection.”.

-----------------------------------------------------------------------------------------------------------------

**Reviewer #2**

Petit and colleagues submitted the manuscript : "Predicting telemedicine use among healthcare professionals in cardiology: a Bayesian model averaging and machine learning analysis of German survey data". Usage of telemedicine in modern medicine is a relevant topic. This manuscript is nicely written in logical order and easy to follow. It analyzes variables that influence implementation of telemedicine in Germany.

However, this title doesn't fully match the text of the manuscript. The authors mentioned machine learning (ML) models such as XGBoost, but the results of the ML testing were not fully analyzed nor explained in details in the manuscript. ML model was not meaningfully incorporated in this study, it's not clear why did authors perform this ML analysis. This study could be completely explained by traditional statistical models. Bayes method is one of them, but significance of these findings is not well explained. If the authors want to keep ML analysis in the text, they need to better elaborate the findings and to compare results with at least 1-2 similar studies where ML models were used.

It's not easy to recognize contribution of this manuscript. Most of findings are well known.
I would suggest the authors to complete ML part with better explanations especially in the discussion section. And comparison of ML findings with other similar studies. Or the alternative is to completely remove the ML study from the text and expand statistical analysis and provide significance of the findings using adequate statistical methods. After correction of the text as suggested they could resubmit the manuscript.

Authors: All changes made to the manuscript are highlighted in yellow in our responses to your comments.

In response to your comment regarding the title, we have revised it to: “Telemedicine adoption in cardiology: determinants and predictors identified using Bayesian model averaging and machine learning” to better reflect the content and analytical approaches presented in the manuscript.

Regarding the machine learning (ML) analysis, we respectfully disagree that traditional statistical models alone would suffice to address the objectives of our study. While Bayesian model averaging (BMA) provides a rigorous inferential framework for identifying determinants and quantifying uncertainty, ML approaches such as XGBoost complement this by offering predictive modeling that can capture complex, potentially non-linear relationships among variables and provide a data-driven assessment of feature importance. These methods serve different purposes: BMA emphasizes explanatory inference, whereas ML emphasizes predictive accuracy and identification of key patterns that may not be evident through traditional modeling. To improve clarity and justify the inclusion of ML in our study, we have extensively revised the manuscript to provide a more detailed description of the ML methodology, the nested cross-validation framework, and the interpretation of SHAP values for predictor importance. We also explicitly discuss how ML findings compare and complement the BMA results.

Please refer to lines 123-149 in the revised version of the manuscript with track changes:

“Different statistical analysis approaches have been used in the past few decades in order to understand the factors associated with TM use. Most previous investigations of TM adoption among HCPs in cardiology-related fields have employed either qualitative methods (19–21), descriptive statistics (22,23) clustering techniques (24,25), or logistic regression models (26–28). While these approaches have provided valuable insights, they also carry limitations, particularly in terms of addressing model uncertainty, achieving robust predictive performance, and capturing complex nonlinear relationships between predictors and outcomes.

In this study, we aimed to address these methodological gaps by applying Bayesian model averaging (BMA) alongside modern explainable machine learning (ML) techniques. BMA is designed to identify determinants that are statistically associated with an outcome (e.g., TM use) and provide insights into potential mechanisms and drivers of behavior. In contrast, ML methods such as extreme gradient boosting (XGBoost), prioritize predictive accuracy and are able to capture complex, non-linear relationships that might remain hidden in conventional regression-based approaches. Unlike conventional regression analyses, which generally identify a single “best-fitting” model and disregard the uncertainty surrounding model specification, BMA formally accounts for this uncertainty by averaging across multiple plausible models weighted by their posterior probabilities (29). This approach yields parameter estimates that are more robust, less sensitive to sampling variability, and therefore more reliable in contexts such as TM adoption, where multiple interdependent factors are at play (17) or ML, we applied XGBoost, which is widely recognized for its strong predictive accuracy, computational efficiency, and capacity to capture nonlinear and high-dimensional dependencies (30–32). XGBoost offers built-in mechanisms to address multicollinearity through hierarchical partitioning and regularization, ensuring stability of the results (30–32).

By combining BMA and ML in the present study, we sought to distinguish between explanatory relevance (factors associated with TM use) and predictive relevance (factors that improve classification of TM users vs. non-users). This dual perspective not only provides complementary insights into the determinants of TM adoption among HCPs but also allows us to assess the robustness of findings across distinct analytical frameworks”.

Please also refer to lines 509-518 in the revised version of the manuscript with track changes (or to lines 509-518 in the revised version without track changes): “All statistical analyses were performed using R software 4.5.0® (R Core Team, Vienna, Austria) for Windows 11©.

*Data management*

Independent variables were defined based on all columns except the outcome variable, TM use. Ordered variables were retained as ordered factors, and nominal categorical variables with more than two levels were one-hot encoded, with one reference category omitted per variable to prevent multicollinearity (21). Missing values were handled as a separate category, under the assumption that nonresponse may reflect systematic differences in TM use. After these steps, the dataset included 53 independent covariates.

*Bayesian model averaging (BMA) analysis*

We implemented a structured three-step pipeline to identify determinants of TM use. First, collinearity screening was conducted by examining pairwise correlations and calculating the variance inflation factor (VIF), with variables exhibiting VIF > 2.5 excluded to mitigate multicollinearity (24) . Next, LASSO regression was applied to reduce dimensionality and retain the most informative variables. Optimal penalization parameters were identified using five-fold cross-validation (CV), and variables with non-zero coefficients at the optimal lambda were retained. These variables were subsequently entered into a BMA framework with logistic regression. BMA averages across all candidate models, weighting by posterior probabilities, thus accounting for model uncertainty (Depaoli et al. 2021). This process provided posterior inclusion probabilities (PIPs) for each feature, indicating the likelihood that the variable has a non-zero effect on TM use. Posterior mean coefficients and standard deviations were calculated across all models (full BMA estimates), and we also summarized results from the single highest posterior probability model and the five highest posterior probability models for comparison. Variables with non-zero PIPs were considered determinants of TM use, with higher PIPs reflecting stronger evidence of association. Visual summaries of group-level differences were produced using radar plots, where continuous and ordinal variables were normalized to a 0–1 range using min–max scaling and summarized by group medians, whereas dichotomous variables were summarized by group proportions.BMA was performed using the BMA package (version 3.18.17) (27). Regarding priors for BMA, we assumed that all candidate models were equally likely a priori (same prior weight).

*Machine learning analysis*

To complement the BMA analysis and leverage the predictive capacity of ML, we applied a binary XGBoost model. Unlike regression models, XGBoost handles multicollinearity through its tree-based structure, so no pre-filtering for collinearity was performed (30–32) To avoid biased evaluation, a nested CV framework was applied. The dataset was first split into a 70% training set and a 30% holdout test set using stratified sampling to preserve the proportion of TM users and non-users. Within the training set, a 10-fold outer CV was performed, with each fold sequentially serving as validation while the remaining folds formed the training subset. Within each outer training fold, a 10-fold inner CV was conducted to tune hyperparameters. Due to computational constraints, 30 random hyperparameter combinations were tested for each fold, with learning rate ([0.05-1]), maximum depth ([3-7]), minimum sum of instance weights (hessian) needed in a child node ([1-10]), subsample ([0.5-1]), and subsample ratio of columns when constructing each tree ([0.5-1]) varied within pre-specified ranges. The scale_pos_weight parameter was set to the ratio of majority to minority class to account for class imbalance. Hyperparameters were optimized by maximizing the mean area under the receiver operating characteristic curve (AUROC) across inner folds. The best hyperparameter configuration for each outer fold was used to train an XGBoost model, which was then evaluated on the corresponding outer validation set.

Following nested CV, the hyperparameter configuration yielding the highest AUROC across outer folds was selected to train a final XGBoost model on the full training set. This model was evaluated on the independent 30% holdout test set. Evaluation metrics included AUROC with 95% confidence intervals, accuracy with 95% confidence intervals, F1-score, and additional metrics such as precision, recall, and specificity. The F1 score is a measure of predictive performance, combining precision and recall into a single value, ranging from 0 (poor performance) to 1 (perfect performance).

The XGBoost algorithm was computed using the xgboost package version 1.7.8.1 (50). The caret package version 7.0-1 was used to calculate the confusion matrix (51). The pROC package version 1.18.5 (52) was used to calculate the AUROC, while the MLmetrics package version 1.1.3 (53) was used to compute evaluation performance metrics not provided by the caret package. The stratified sampling was performed with the splitTools package version 1.0.1 (54) . Model training, cross-validation, and hyperparameter tuning were carried out with the mlexperiments package (55) in combination with the mllrnrs package (56), which provide standardized machine learning pipelines and learner interfaces.

To enhance the interpretability of the XGBoost model, SHapley Additive exPlanations (SHAP) values were calculated using the holdout test set to quantify the contribution of each predictor to the model's output. SHAP values reflect the individual contribution of each predictor by evaluating its marginal impact on the model’s output (57,58).Predictors that directed the model toward TM use were considered promoting factors, whereas predictors directing the model toward non-use were considered limiting factors. Directionality was inferred using a hybrid heuristic. For binary and sparse features, mean SHAP values were compared across groups, while for continuous features, generalized additive models (GAMs) were fitted to SHAP values, and the average derivative of the fitted curve was used to determine association direction. When GAMs failed to converge, Spearman’s rank correlation between the feature and its SHAP values served as a fallback.

*Sensitivity analysis*

A sensitivity analysis was performed by excluding individuals with missing values to evaluate the robustness of our findings.”.

Please also refer to lines 509-518 in the revised version of the manuscript with track changes (or to lines 509-518 in the revised version without track changes): “*BMA analysis*

Of the 53 independent covariates initially considered, 32 (60%) were retained after excluding collinear variables. Among these 32, 12 (38%) were selected using Least Absolute Shrinkage and Selection Operator (LASSO) regression and subsequently included in the BMA analysis. Examination of posterior model probabilities revealed model uncertainty. No single model dominated, with the highest posterior model accounting for only 11.0% of the total weight, and the top five models together representing 41.3%. Estimates based on only the top model, or approximated across the top five models, differed from the fully model-averaged estimates (Table 1, Figure 1). In contrast, posterior means from the full BMA, which incorporate information from the entire model space, provided more stable estimates that were often shrunk toward zero.

**Figure 1**: Coefficient estimates for each predictor variable under three modeling perspectives: the highest posterior probability single model (“Top model”), an approximation averaged over the top five models (“Top 5 models”), and the fully Bayesian model-averaged estimates (“BMA”). The points represent the estimated coefficient effect sizes, while the error bars show the posterior standard deviations derived from the full BMA analysis, reflecting uncertainty in these averaged estimates. The “Top model” and “Top 5 models” estimates do not include uncertainty intervals because these approximations do not capture model-averaged uncertainty. The full “BMA” estimates incorporate all plausible models weighted by their posterior probabilities, shrinking coefficients toward zero when evidence for inclusion is weak and properly reflecting parameter uncertainty.

A total of 12 determinants of TM use were identified (Table 1, Figure 2). Positive associations were found for TM knowledge, being a cardiologist, self-identifying as female, perceiving TM as suitable for heart failure and for monitoring an event, considering absence of remuneration and data security as barriers to TM, and perceiving TM as relevant for extending the aftercare interval and for basic ECG monitoring. Negative associations were observed for considering insufficient data on patient benefit as a TM barrier, and for perceiving TM as less suitable in acute events or for extending the aftercare interval (Table 1).

The sensitivity analysis produced similar findings, identifying eight determinants, all of which overlapped with the main analysis and showed consistent directions of association (Figure 2, Table S1, Figure S1). However, in this analysis, data security, the relevance of TM for ECG monitoring and aftercare extension, and the unsuitability of TM in acute events were not retained as determinants.

**Table 1**: Factors Identified Through BMA and Their Association With TM Use

The determinants identified through BMA were used to describe the profiles of HCPs in cardiology who use and do not use TM (Figure 2). Profiles are presented for both the main and sensitivity analyses. The percentages correspond to the proportion of HCPs endorsing each specified response. For example, 100% (the outer line of the radar chart) indicates that all HCPs selected the given response (e.g., self-identifying as female), whereas 0% (the inner line) indicates that none selected it.

**Figure 2**: Profile of healthcare professionals (HCPs) in cardiology using telemedicine (TM) versus non-users. The radar plot compares key determinants, with TM users (green) and non-users (red).

*Machine Learning analysis*

The XGBoost model demonstrated strong performance, with an AUROC of 0.88 (95% CI: 0.75–1.00), an accuracy of 0.79 (95% CI: 0.62–0.91), a sensitivity of 79%, a specificity of 80%, and an F1 score of 0.81 (Table 2).

**Table 2**: Performance of the final XGBoost model

***Note***: The purpose of the McNemar’s test is to test whether the number of false positives and false negatives are statistically equal.

Several of the most influential predictors identified by SHAP analysis (e.g., TM knowledge, being a cardiologist, and female sex) were also among the key determinants highlighted in the BMA analysis. Figure 3, Table 3). The ML framework identified additional predictors that did not emerge as determinants in the BMA models. For example, perceptions of TM suitability for arrhythmias, pacemaker and defibrillator therapy, and for monitoring blood pressure ranked highly in predictive importance, reflecting the model’s ability to capture more complex or non-linear associations. Conversely, factors such as concerns about insufficient patient benefit, which were negatively associated with TM use in BMA, did not contribute substantially to the predictive performance of the ML model.

In terms of directionality, SHAP values indicated that several factors promoted TM use, including TM knowledge, being a cardiologist, female sex, perceiving TM as suitable for arrhythmias and heart failure, higher patient volume per quarter, and viewing TM as relevant for managing complications of drugs and medical devices. In contrast, limiting factors included perceiving TM as unsuitable for acute events or aftercare extension, older age, using TM for own health, and concerns about implementation effort (Table 3, Figure 3).

The sensitivity analysis yielded broadly consistent findings, although some predictors identified in the main model were not retained, such as perceptions of insufficient remuneration as a barrier or suitability for hypertension (Table 2, Figure S2, Table S2). At the same time, new predictors emerged, such as the perception that TM is less suitable for basic ECG monitoring or for consulting colleagues. Certain predictors shifted directionality. For instance, the number of patients per quarter and willingness to invest in TM showed opposite associations compared with the main model. These discrepancies emphasize that while the overall predictive structure of the ML model was stable, the importance of individual features may vary depending on the population considered.

**Figure 3**: Feature importance according to SHAP values for the prediction of TM use.

SHAP value (x-axis) for each HCP and feature are represented with a point. Positive SHAP values imply an impact to the model toward wanting TM use, while negative values impact the model toward not using TM. For each feature, the mean and 95% CI of the absolute SHAP values are reported on the right of the graph. High SHAP value (in absolute value) indicate a high impact on the model output. Red colors indicate that an HCP either answered yes to the considered question (y-axis) regarding categorical feature or tend toward high values for continuous features (e.g., age), while blue colors refer either to “no” answers for categorical features or low values for continuous features, and grey colors refer to missing answers.

**Table 3**: Top predictors of TM Use identified with XGBoost

*BMA and XGBoost comparison*

The explanatory and predictive frameworks converged on several key factors while also highlighting different aspects of TM adoption (Table 4). Both BMA and XGBoost identified TM knowledge, female sex, and being a cardiologist as robust determinants of TM use. These variables demonstrated both a consistent statistical association and a strong contribution to predictive performance, suggesting that they represent core drivers of adoption in cardiology practice. Similarly, perceptions that TM is unsuitable for acute events or for extending aftercare intervals were negatively associated with use in BMA and also reduced predictive probability in XGBoost, reinforcing their importance as barriers.

Beyond these points of convergence, the two approaches diverged in meaningful ways. BMA, by design, emphasized parsimonious associations with stable posterior inclusion probabilities, highlighting concerns about insufficient patient benefit as a limiting factor, an effect that did not substantially improve the predictive capacity of the XGBoost model. Conversely, XGBoost identified perceptions of TM suitability for arrhythmias, device monitoring, and blood pressure management as strong predictors, even though these factors did not consistently emerge as explanatory determinants in BMA. These differences underscore the complementary nature of the two methods. BMA provides insights into determinants with stable associations, while ML captures a broader set of variables that enhance classification accuracy, including those with weaker or more context-dependent effects.

Importantly, one feature (perceptions of TM suitability for event monitoring) exhibited incongruent behavior across approaches, showing a positive association in one framework (BMA) but a limiting effect in the other (XGBoost). Such discrepancies highlight the value of applying both explanatory and predictive methodologies, as they draw attention to variables whose influence on TM adoption may depend on context, measurement, or modeling assumptions.

**Table 4**: Comparison of the feature impact on TM use depending on the statistical method use

***Note***: empty cells refer to features that were not selected in the models.”.

Please also refer to lines 509-518 in the revised version of the manuscript with track changes (or to lines 509-518 in the revised version without track changes): “This study applied BMA and XGBoost to move beyond confirming general associations by estimating the relative importance of predictors, producing more robust effect estimates, and exploring potential non-linear relationships to provide a more nuanced picture of TM adoption among HCPs. The findings can be summarized as follows. First, across both frameworks, TM knowledge, female sex, and being a cardiologist consistently emerged as core drivers of TM use. Second, the lack of remuneration was identified as a main barrier for TM use in both models. Third, the two approaches also highlighted distinct aspects of TM adoption: BMA emphasized concerns about insufficient patient benefit as a limiting factor, whereas XGBoost identified perceptions of TM suitability for arrhythmias, device monitoring, and blood pressure management as strong predictors. This complementarity underscores the value of combining explanatory and predictive perspectives, explanatory models reveal stable associations that clarify underlying mechanisms, while predictive models capture additional and sometimes non-linear factors that improve classification.”.

Please refer to lines 123-149 in the revised version of the manuscript with track changes

Different statistical analysis approaches have been used in the past few decades in order to understand the factors associated with TM use. Most previous investigations of TM adoption among HCPs in cardiology-related fields have employed either qualitative methods (19–21), descriptive statistics (22,23) clustering techniques (24,25), or logistic regression models (26–28). While these approaches have provided valuable insights, they also carry limitations, particularly in terms of addressing model uncertainty, achieving robust predictive performance, and capturing complex nonlinear relationships between predictors and outcomes.

In this study, we aimed to address these methodological gaps by applying Bayesian model averaging (BMA) alongside modern explainable machine learning (ML) techniques. BMA is designed to identify determinants that are statistically associated with an outcome (e.g., TM use) and provide insights into potential mechanisms and drivers of behavior. In contrast, ML methods such as extreme gradient boosting (XGBoost), prioritize predictive accuracy and are able to capture complex, non-linear relationships that might remain hidden in conventional regression-based approaches. Unlike conventional regression analyses, which generally identify a single “best-fitting” model and disregard the uncertainty surrounding model specification, BMA formally accounts for this uncertainty by averaging across multiple plausible models weighted by their posterior probabilities (29). This approach yields parameter estimates that are more robust, less sensitive to sampling variability, and therefore more reliable in contexts such as TM adoption, where multiple interdependent factors are at play (17) or ML, we applied XGBoost, which is widely recognized for its strong predictive accuracy, computational efficiency, and capacity to capture nonlinear and high-dimensional dependencies (30–32). XGBoost offers built-in mechanisms to address multicollinearity through hierarchical partitioning and regularization, ensuring stability of the results (30–32).

By combining BMA and ML in the present study, we sought to distinguish between explanatory relevance (factors associated with TM use) and predictive relevance (factors that improve classification of TM users vs. non-users). This dual perspective not only provides complementary insights into the determinants of TM adoption among HCPs but also allows us to assess the robustness of findings across distinct analytical frameworks

-----------------------------------------------------------------------------------------------------------------

**Reviewer #3**

The paper presents a secondary analysis of survey data from German healthcare professionals to determine the factors that influence and forecast the use of telemedicine in cardiology. The work is technically sound and provides insightful information about the uptake of digital health from a data science standpoint. Below are my suggestions to the authors to improve the paper:

1. To meet PLOS's data sharing guidelines, think about storing anonymised survey data in a public repository.

Authors: Thank you for your review and your relevant comments. We value your time and effort. All changes made to the manuscript are highlighted in yellow in our responses to your comments. All R scripts used for analysis have been made publicly available, as detailed in the Data Availability Statement. Although our dataset stems from an anonymous survey, the combination of detailed sociodemographic and professional characteristics could potentially allow for the re-identification of individual respondents. Importantly, our institutional ethics committee explicitly approved the study under the condition that no individual-level survey data would be made publicly available, and participants did not provide consent for such public data sharing. Consequently, depositing the dataset in an open repository is not possible. However, the dataset can be made available upon reasonable request to the corresponding author, provided that appropriate data protection measures are in place.

2. The rigor section would be strengthened by a brief explanation of possible overfitting hazards despite nested cross-validation, given the relatively small sample size (n=112) and large number of initial variables (n=53).

Authors: We followed your suggestion. Please refer to lines 509-518 in the revised version of the manuscript with track changes (or to lines 509-518 in the revised version without track changes): “We further explored variable relevance by applying an explainable ML approach, combining XGBoost, a powerful algorithm widely applied in predictive modeling tasks (37, 38), with SHAP to enhance interpretability. The use of nested cross-validation represents a methodological strength, as it separates hyperparameter tuning from model evaluation and thereby reduces the risk of overfitting. Nevertheless, despite this safeguard, the relatively small sample size (n=112) and the high number of initial variables (n=53) could still allow the model to capture dataset-specific patterns, potentially inflating variable importance. The model’s strong predictive performance highlights the relevance of the identified factors and suggests that targeted interventions, such as structured training and administrative reforms, could effectively address adoption barriers and support broader TM integration in clinical practice. Although we did not validate our model using an independent dataset or multiple resampling iterations, the nested CV framework provides a reliable estimate of predictive performance and helps ensure that the identified predictors are supported within the training data. Our dataset includes a heterogeneous group of HCPs from diverse clinical settings, supporting the relevance of the findings. However, generalizability remains limited by the relatively small sample size, geographic restriction to two regions in Germany, and absence of external validation. Future studies should aim to replicate and extend this predictive framework using larger, independent datasets to assess its applicability across broader healthcare contexts.”.

3. Although the authors point out that there isn't an independent test set, the significance of the paper would be increased with a clear plan for future validation.

Authors: We followed your suggestion.

Please refer to lines 509-518 in the revised version of the manuscript with track changes (or to lines 509-518 in the revised version without track changes): “Future studies should aim to replicate and extend this predictive framework using larger, independent datasets to assess its applicability across broader healthcare contexts.

A key limitation of our study is the cross-sectional nature of the survey data, which limits causal interpretation. The aim of this study was not to establish predictive causality, but rather to explore and characterize distinct profiles of TM users. Some of the variables included as predictors, such as TM knowledge, perceptions of TM suitability for heart failure or arrhythmias, experience with cardiac devices, and considerations of remuneration, reflect current practices and attitudes rather than factors that necessarily preceded TM adoption. As such, the predictive models in this study estimate associations with existing TM use, rather than truly forecasting future adoption. A longitudinal design, in which TM use is measured prospectively, would allow for a more rigorous evaluation of causal relationships and the development of predictive models that identify determinants prior to adoption. Future research should validate our findings in independent and temporally structured datasets, ideally with repeated measures of TM use and predictors over time. Such studies would enable more accurate prediction of TM adoption and help distinguish factors that actively drive uptake from those that are concomitant with current usage”.

4. While handling missing data as distinct categories makes sense, a sensitivity analysis could bolster robustness assertions even further.

Authors: We followed your suggestion by conducting a sensitivity analysis excluding individuals with missing values to evaluate the robustness of our findings. This sensitivity analysis yielded similar findings than the main analysis.

Please refer to lines 509-518 in the revised version of the manuscript with track changes (or to lines 509-518 in the revised version without track changes): “The sensitivity analysis produced similar findings, identifying eight determinants, all of which overlapped with the main analysis and showed consistent directions of association (Figure 2, Table S1, Figure S1). However, in this analysis, data security, the relevance of TM for ECG monitoring and aftercare extension, and the unsuitability of TM in acute events were not retained as determinants.”.

Please also refer to lines 509-518 in the revised version of the manuscript with track changes (or to lines 509-518 in the revised version without track changes): “The sensitivity analysis yielded broadly consistent findings, although some predictors identified in the main model were not retained, such as perceptions of insufficient remuneration as a barrier or suitability for hypertension (Table 2, Figure S2, Table S2). At the same time, new predictors emerged, such as the perception that TM is less suitable for basic ECG monitoring or for consulting colleagues. Certain predictors shifted directionality. For instance, the number of patients per quarter and willingness to invest in TM showed opposite associations compared with the main model. These discrepancies emphasize that while the overall predictive structure of the ML model was stable, the importance of individual features may vary depending on the population considered.”.

Please also refer to lines 509-518 in the revised version of the manuscript with track changes (or to lines 509-518 in the revised version without track changes): “*Sensitivity analysis*

A sensitivity analysis was performed by excluding individuals with missing values to evaluate the robustness of our findings.”.

Please also refer to the Supplementary materials.

5. Although figures are instructive, interpretability might be improved by including confidence intervals for SHAP value ranks or by including an additional plot that illustrates feature interaction effects.

Authors: The SHAP values presented in Figure 3 and Table 3 already include confidence intervals, providing an estimate of uncertainty around each predictor’s contribution. This allows readers to assess the robustness of the feature rankings. While we did not generate additional plots illustrating feature interactions, the confidence intervals in the current presentation capture the variability of individual feature effects across the holdout test set.
